# Parallel declines in species and genetic diversity driven by anthropogenic disturbance: a multispecies approach in a French Atlantic dune system

**DOI:** 10.1111/eva.12351

**Published:** 2016-01-27

**Authors:** David Frey, Nils Arrigo, Gilles Granereau, Anouk Sarr, François Felber, Gregor Kozlowski

**Affiliations:** ^1^ Natural History Museum of FribourgFribourgSwitzerland; ^2^ Department of Biology and Botanical GardenUniversity of FribourgFribourgSwitzerland; ^3^ Department of Ecology and EvolutionUniversity of LausanneLausanneSwitzerland; ^4^ Office national des forêts, réseau habitats – florePouillonFrance; ^5^ Laboratory of Evolutionary BotanyInstitute of BiologyUniversity of NeuchâtelNeuchâtelSwitzerland; ^6^ Musée et Jardins botaniques cantonauxLausanneSwitzerland

**Keywords:** biodiversity, coastal conservation, disturbance, habitat loss, sand dunes, species–genetic diversity correlation metapopulation model, urbanization

## Abstract

Numerous studies assess the correlation between genetic and species diversities, but the processes underlying the observed patterns have only received limited attention. For instance, varying levels of habitat disturbance across a region may locally reduce both diversities due to extinctions, and increased genetic drift during population bottlenecks and founder events. We investigated the regional distribution of genetic and species diversities of a coastal sand dune plant community along 240 kilometers of coastline with the aim to test for a correlation between the two diversity levels. We further quantify and tease apart the respective contributions of natural and anthropogenic disturbance factors to the observed patterns. We detected significant positive correlation between both variables. We further revealed a negative impact of urbanization: Sites with a high amount of recreational infrastructure within 10 km coastline had significantly lowered genetic and species diversities. On the other hand, a measure of natural habitat disturbance had no effect. This study shows that parallel variation of genetic and species diversities across a region can be traced back to human landscape alteration, provides arguments for a more resolute dune protection, and may help to design priority conservation areas.

## Introduction

The analogy between the processes determining genetic diversity (GD) within species and species diversity (SD) within communities has long been recognized (Antonovics 1976). It has been hypothesized that the environment might influence biodiversity at various levels in parallel, resulting in positive correlations among these levels (Vellend and Geber 2005). Indeed, several empirical studies detected positive GD–SD correlations (hereafter ‘SGDC’, Vellend 2003, 2004; Cleary et al. 2006; Papadopoulou et al. 2011; Struebig et al. 2011; Blum et al. 2012; Wei and Jiang 2012; Kahilainen et al. 2014); however, only a handful of studies have explored the processes underlying the observed patterns (Lamy et al. 2013; Vellend et al. 2014; Laroche et al. 2015).

At the regional scale and over short evolutionary scales, selectively neutral GD within a population is driven by demographic processes such as migration and genetic drift (Hartl and Clark 1997). These processes are in turn tightly linked to habitat characteristics (Vellend and Geber 2005). For example, landscape fragmentation and habitat disturbance may decrease migration rates among local populations, introduce abrupt population size reductions, or even cause local extinctions. Such events will decrease GD via increased genetic drift (e.g., Slatkin 1977; Banks et al. 2013). In addition, while disturbance might create colonization opportunities, genetic bottlenecks associated with the re‐colonization process further delay the restoration of GD (Whitlock and McCauley 1990; Banks et al. 2013). SD within a local community might respond similarly to habitat disturbance (Vellend and Geber 2005). Indeed, disturbance may wipe out local populations of a species or reduce census size, which increases local extinction risks due to environmental, demographic and genetic stochasticity, and recurring disturbance events (e.g., Townsend 1989; Primack 1995).

At the community level, it thus appears that GD and SD respond in parallel to habitat disturbance (see Box 1 for a simulation‐based example). If disturbance varies within a region, regional SGDC patterns may emerge. However, this correlation decreases when species differ in their reactions to environmental variation or if they compete with each other. In addition, GD patterns depend on how fast a migration–drift equilibrium is reached in a given context. Accordingly, fast‐mutating markers – such as SSRs – will reveal SGDCs over a short evolutionary time frame. In contrast, slowly evolving markers will retain historical footprints (e.g., phylogeographic differentiation and range expansion patterns left by ancient glacial events) that are independent of ongoing habitat‐driven processes and will be of limited use to uncover SGDCs (Vellend 2004; Wehenkel et al. 2006; Derry et al. 2009; Odat et al. 2010).

Box 1 **SD and GD react similarly to environmental disturbance: a simulation‐based example**

For illustrative purposes, we implemented a metapopulation model to assess how habitat disturbance impacts genetic and community diversity. In our simulations, habitat disturbance is the major driver of the extinction–recolonization dynamics of all the species in the community (panel A, all species share the same probability of encountering a disturbance event). By promoting founder effects and driving local resampling of alleles and species, habitat disturbance decreases both genetic and community diversity (panel B) and ultimately creates SGDCs (panel C). This mechanism, coupled with migration among patches, promotes SD–GD correlations in various phylogeographic contexts, provided that there is sufficient time to allow the system to reach a migration–drift equilibrium. In our example simulation, it takes only 200 generations to wipe out any initial phylogeographic background and generate a consistent SGDC pattern (panel C). These results are robust to interspecies variations in key life‐history traits such as sensitivity to disturbance events, mating systems, migration abilities, and effective population sizes. The interested readers are referred to the recent works of Laroche et al. (2015) for a complete theoretical treatment of SGDCs in a neutral theory framework.

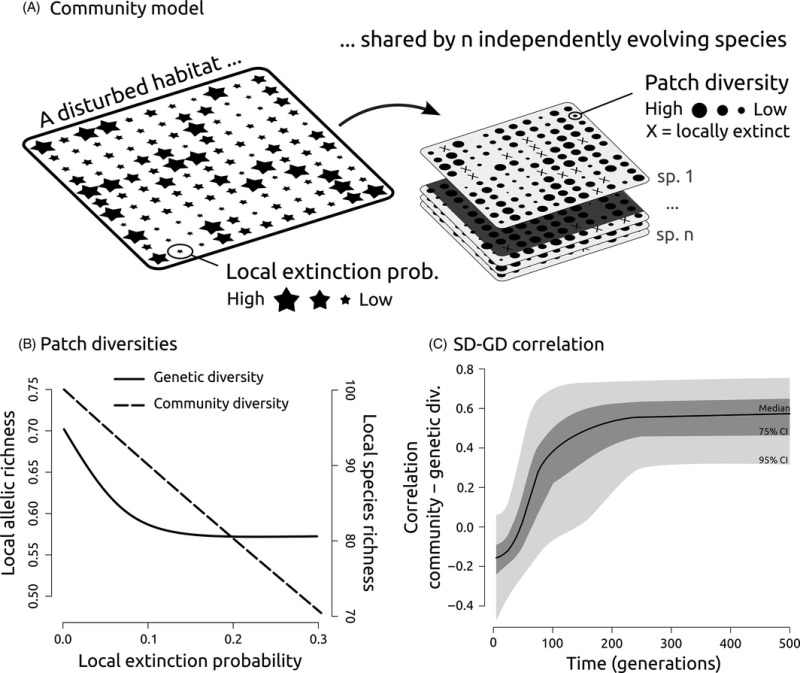

Metapopulation model simulating how habitat disturbance jointly impacts genetic and community diversity. We consider an 11 × 11 landscape of interconnected patches (represented as stars that experience varying disturbance regimes, implemented here by modulating local extinction probabilities, represented as stars of varying diameters). This disturbed landscape is then used to simulate the evolution of 100 independent species for 500 generations until the system reaches a migration–drift equilibrium and the initial conditions have no further effect. Diversity statistics represented as dots of varying diameter are collected in each patch over time by measuring (i) the average genetic diversity (i.e., allelic richness across all species) and (ii) the number of species occupying each patch (i.e., species richness). The collected diversity estimates are then related to local extinction probabilities, and their pairwise correlation is tracked through generations. B Diversity estimates related to local extinction probabilities, as observed when the system is at equilibrium (500 generation). C Correlation between genetic and community diversity (SD–GD correlation as a function of time). The median straight line and 75 % and 95 % confidence interval envelopes are displayed. These results are robust to interspecies variations in life‐history traits (i.e., varying effective population sizes, selfing rates, migration rates, and reactions to extinction and historical backgrounds (i.e., starting metapopulations with a random genetic makeup or a strong initial phylogeographic structure). B and C are based on 100 replicates. Migration events are allowed only among adjacent patches. Extinction probabilities are constant through time. All simulations were performed using QuantiNemo (Neuenschwander et al. 2008).

Positive SGDCs are mainly expected in communities in which most species react in a similar way to one dominant environmental factor and in which interspecific competition is relatively low (Vellend and Geber 2005). The vegetation of coastal sand dunes may be such a community. On dunes, SD is largely predicted by the disturbance regime, with a general trend toward higher SD under lower disturbance intensity and frequency (García–Mora et al. 2000; Forey et al. 2008; Maun 2009). Disturbance on dunes includes mainly burial by sand and substrate erosion, which can be triggered by other factors such as the destruction of vegetation cover by trampling, storm surges, and wave scarping (e.g., Lemauviel and Rozé 2003; Hesp and Martínez 2007). Although disturbance is frequent on dunes and is part of the natural dune dynamics, excessive disturbance can lead to the complete loss of vegetation followed by dune erosion, which can have long‐lasting negative effects on SD (Rozé and Lemauviel 2004; Thompson and Schlacher 2008).

Increased disturbance leading to the erosion and fragmentation of coastal sand dunes mainly occurs due to human activities and represents a worldwide trend (Martínez et al. 2007, 2008). Indeed, global change occurs faster in coastal areas and where the human population density is disproportionally high (Martínez et al. 2007). For example, the French coast represents only 4% of the national territory but is home to 6 million people and has accommodations sufficient to support 7 million visitors (Observatoire du littoral 2013).

Hence, many coastal dune systems are degrading, and numerous native plant species are declining (Van der Maarel and Van der Maarel‐Versluys 1996; Schlacher et al. 2007; Martínez et al. 2008; Defeo et al. 2009). From a conservation perspective, this process implies an important loss of natural resources because coastal dunes can harbor a large number of narrowly endemic plants (Van der Maarel and Van der Maarel‐Versluys 1996; Harris et al. 2014) and may contribute to important ecosystem services such as coastal protection by dune plants (Martínez et al. 2007).

In this study, we assess the regional distribution of GD and SD in a coastal sand dune plant community along 240 km of the Atlantic shoreline in southwestern France. To this end, we use a comprehensive ecological survey and genotype seven representative plant species with amplified fragment length polymorphisms (i.e., AFLPs are considered effectively neutral and have been widely used to detect signatures of genetic drift and loss of genetic diversity; Bonin et al. 2007). We then assess whether regional variations in GD and SD are correlated with each other and ask whether habitat disturbance – of natural and/or anthropogenic origin – could provide the underlying mechanism that explains the observed pattern.

## Methods

### Study area and sand dune habitat

**Figure 1:**
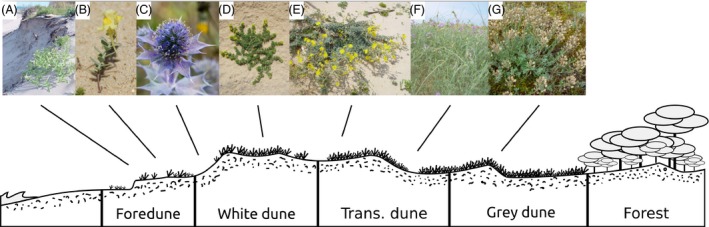
Simplified dune profile showing the four main vegetation zones (i.e., dune facies of the French Atlantic coast (Favennec 2002a) arranged between the sea and the forest line); pictures of the species used for genetic analysis positioned according to their preferred habitat (A, *Cakile maritima*; B, *Linaria thymifolia*; C, *Eryngium maritimum*; D, *Galium arenarium*; E, *Hieracium eriophorum*; F, *Astragalus baionensis*; G, *Alyssum loiseleurii*).

The Atlantic shoreline of southwestern France shelters the longest coastal sand dune in Europe. This ecosystem represents a large and uninterrupted sand belt of ca. 240 km. It is 200–500 m wide and is delimited at its northern and southern edges by river mouths and bedrock outcrops; landwards, it is delimited by coastal forests (Favennec 2002a). The coastal dunes harbor a highly adapted plant community of approximately 200 species (Favennec 1998). Eleven of these are endemic to the French Atlantic coast and occur mainly in the study area (e.g., Van der Maarel and Van der Maarel‐Versluys 1996; Favennec 1998). The dune vegetation follows a typical beach inland succession pattern (i.e., dune facies; Forey et al. 2008; Maun 2009), coinciding with increasing species richness and vegetation cover (Géhu and Géhu 1969; Favennec 2002a; Fig. 1). This gradient also correlates with sand erosion and deposition dynamics and reflects natural levels of habitat disturbance (Forey et al. 2008; Maun 2009, see below for a quantitative treatment).

We investigated the sand dune plant community by combining three lines of evidence: (i) a detailed ecological survey of sand dune habitats to assess species diversity, (ii) a genetic analysis of seven representative plant species, and (iii) a detailed ecological survey to assess the drivers of SGDCs (with respect to natural and anthropogenic habitat disturbance). These three datasets were initially collected at distinct spatial resolutions and were all averaged over the same 5‐km‐resolution grid prior to statistical analysis.

### Ecological survey: species diversity and natural habitat disturbance

The study area was surveyed during June 2003 by the French National Forest Office (ONF) over 773 circular plots (100 m^2^ each) arranged along 94 geo‐referenced transects (on average, one transect every 2.5 km) that spanned the beach inland gradient. Plant species composition and a comprehensive set of habitat characteristics (Fig. S1) were recorded within each plot, allowing a detailed characterization of the community diversity at each surveyed site.

Species diversity was assessed at the transect level to account for the entire sand dune community. Invasive and weedy plants (14) unspecific to the sand dune plant community (Favennec 1998) were excluded from the calculations [however, their inclusion did not affect the final results, Tables S4 and S5]. First, we counted the number of distinct species per transect, yielding the species diversity index *S*. Second, we refined this statistic by calculating the proportion of species considered endemic to the French Atlantic coast within each transect (Van der Maarel and Van der Maarel‐Versluys 1996; Favennec 1998), yielding the endemism rate *E*.

Natural disturbance was quantified using a principal component analysis computed at the ONF sampling plot scale and based on eight representative environmental descriptors (detailed in Fig. S1): (i) dune facies (Fig. 1), (ii) exposure, (iii) litter amount, (iv) microtopography, (v) rabbit activity, (vi) ranged distance of sampling plots from forest to ocean, (vii) slope, and (viii) wind perturbation regime. The first PCA eigenaxis, explaining 13% of the total variance, was strongly correlated with the beach inland disturbance gradient typical of coastal ecosystems and was used as a synthetic descriptor of the natural disturbance observed in each ONF sampling plot (a similar procedure was applied by Lamy et al. 2013; see Fig. S1). Hence, positive values described disturbed plots (closer to the ocean and with low vegetation cover), whereas negative values described stable areas (closer to the forest and with high vegetation cover; see Fig. S1). This estimate was further averaged over a 5‐km‐resolution grid (hereafter *H*) to reflect regional variations in natural disturbance.

### AFLP survey of genetic diversity

**Figure 2:**
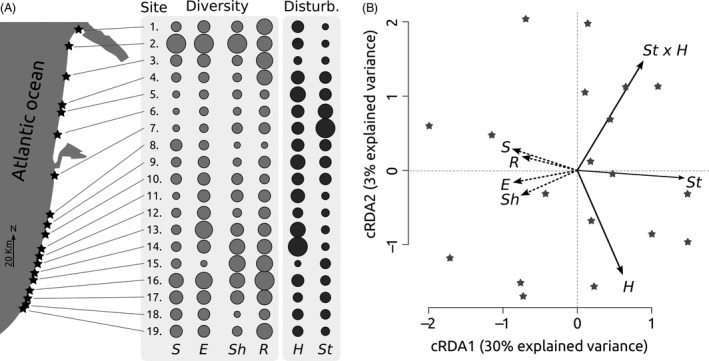
(A) Right panel: Map showing the coastline of the study region, with sampling sites displayed as stars. Left panel: The corresponding community (species diversity, *S*; endemism rate, *E*)*,* genetic diversity (average standardized genetic diversity across seven plant species, as estimated by the Shannon index, *Sh,* and by the rarity index, *R*) and habitat disturbance regime (i.e., natural dune dynamic, *H*; anthropogenic pressure, *St*) are shown as circles (with diameters quantitatively proportional to the focal variable). The effects of spatial autocorrelation have been removed. (B) Partial constrained redundancy analysis. This multivariate approach allows visualization, quantification, and testing (see Table 2 of how habitat disturbance (*H*,* St,* and their interaction *St *×* H* accounts for local variations in species and genetic diversity (*S*,* E*,* Sh,* and *R*, while removing the effects of spatial autocorrelation. Habitat disturbance accounts for 34% of the variance in diversity statistics. The dependent and explanatory variables are represented using dashed and straight lined arrows, respectively. The sampling sites are displayed as stars.

We visited 26 sites neighboring those included in the ONF ecological survey during June 2009 to survey the genetic diversity of the plant community. To this end, we collected a total of 290 specimens across seven regionally characteristic and abundant dune plants (five specimens per species and per site, Fig. 2A and Table S1). These species include five endemic (*Alyssum loiseleurii –* Brassicaceae, *Astragalus baionensis* – Fabaceae, *Galium arenarium –* Rubiaceae, *Hieracium eriophorum –* Asteraceae, and *Linaria thymifolia –* Plantaginaceae) and two widespread species (*Cakile maritima –* Brassicaceae and *Eryngium maritimum –* Apiaceae) (Favennec 1998). DNA was extracted from silica gel‐dried leaves using a CTAB protocol (Doyle and Doyle 1987). AFLPs were generated following protocols detailed in Arrigo et al. (2011) and Frey et al. (2012a) using two to three primer pairs (Table S2) per species [note that the AFLP dataset of *H. eriophorum* was from Frey et al. (2012a)]. The complete protocol (from DNA extraction to genotyping) was replicated for 15% of the specimens to assess the reproducibility of the results (Bonin et al. 2004). The AFLP bands were scored using RawGeno, an R CRAN library (Arrigo et al. 2009), following recommendations made by Arrigo et al. (2012).

Regional genetic diversity was assessed using a ‘moving window’ approach (Arrigo et al. 2010; scripts available upon request) considering the 5‐km‐resolution grid defined earlier. In each cell, diversity levels were estimated with the Shannon information content, a non‐model‐based estimator frequently applied to dominant markers, and with the rarity index, an estimator based on the frequency of rare alleles within populations (Schönswetter and Tribsch 2005; Bonin et al. 2007). Both diversity statistics were computed using a rarefaction procedure to account for unequal sample size among sites (four specimens randomly chosen in each cell, with final diversity estimates averaged over 1000 jackknife resamplings). Finally, regional genetic diversity metrics were first obtained separately for each analyzed species (Fig. S2) before being averaged (after standardization, as in Taberlet et al. 2012) into synthetic estimates (hereafter, *Sh* and *R*) to be used in further analyses.

### Anthropogenic disturbance

Anthropogenic disturbance was estimated from publicly available data (French National Sea and Coast Observatory; ONML) describing the amount (in hectares) of urbanized area within 10 km of the coastline in our study region (Table S3). This descriptor is very highly correlated with the resident population census, the number of houses, and tourist accommodation capacity (Table S3) and was used here as a surrogate of anthropogenic habitat disturbance (hereafter, *St*). It is highly variable along the coastline, ranging from 29.01 to 3278.75 hectares (Table S3). As above, *St* was averaged over the same 5‐km‐resolution grid.

### Statistical analyses: SGDCs and association with habitat disturbance

We merged the SD, GD, natural, and anthropogenic disturbance estimates into a single matrix by considering shared 5‐km grid cells among datasets (resulting in 19 shared grid cells [Fig. 2]).

The association between SD and GD was assessed using pairwise Pearson's correlations. First, we corrected diversity indexes for spatial autocorrelation, to improve the statistical independence of our 19 data points and enhance the accuracy of our statistical tests, by (i) regressing each variable (*S*,* E*,* Sh*,* R*) against the latitudinal coordinates of each grid cell and (ii) extracting residuals for measuring pairwise correlations among diversity estimates. Significance levels were then evaluated using 50 000 randomized permutations. The procedure was performed using custom R scripts (available upon request).

The respective effects of natural and anthropogenic disturbance on diversity levels were assessed using a correlation decomposition procedure (as in Lamy et al. 2013, Data S1). Briefly, this analysis quantifies how the predictors of interest contribute to SGDCs, by introducing environment‐driven and covarying responses in the SD and GD datasets. Tests of statistical significance were performed with a partial constrained redundancy analysis (as implemented in the vegan R CRAN package). This multivariate approach allows visualization, quantification, and testing of how habitat disturbance (i.e., *H*,* St* and their interaction *St *×* H*) accounts for local variations in species and genetic diversity (*S*,* E*,* Sh* and *R*) while removing the effects of spatial autocorrelation by considering the latitudinal coordinates of each grid cell as a covariate. The analysis was performed on standardized data, and significance levels were assessed using 50 000 random permutations.

## Results

The ONF flora survey revealed the presence of 129 different plant species. Throughout the study area, diversity estimates varied from 2.8 to 33.7 species and 4.7 to 13.4 endemics per grid cell. The AFLP analysis produced a total of 555 polymorphic bands with an average of 80 bands per species and a median error rate of 4.36% (Table S2). Within all seven species, GD estimated by the Shannon information content and the rarity index varied markedly among grid cells (Fig. S2), with estimates of *Sh* ranging between 0.06 and 0.23 and estimates of *R* ranging between 0.54 and 2.93.

The flora and AFLP surveys revealed a strong SGDC, with *S*,* E*,* Sh,* and *R* being significantly correlated with each other (Table 1). Accordingly, both datasets showed that the highest diversity levels were found in the southernmost and the northernmost parts of the study region. In addition, a clear diversity decrease, extending over sampling sites 3–8 (at latitudes ranging between 44.2° and 44.8°), was revealed by the flora and AFLP surveys (Fig. 2A).

**Table 1:** Correlation between species (SD) and genetic diversity (GD) estimated for 19 geographical grid cells sampled along the Atlantic coast. The contribution of explanatory variables (natural dune dynamic – H; anthropogenic pressure – St and their interaction – St* *×* *H) to each pairwise correlation is provided

Diversity comparison†	Correlation‡	Contrib. *H* § (%)	Contrib. *St* §(%)	Contrib. St ×* H* §(%)	Contrib. Residuals§(%)
*S* – *Sh*	0.51*	0.01 (2.54)	0.15 (29.81)	0.01 (1.03)	0.34 (66.61)
*S* – *R*	0.42*	0.08 (18.24)	0.18 (43.13)	0.01 (2.42)	0.15 (36.21)
*E* – *Sh*	0.50*	−0.01 (−1.62)	0.20 (40.59)	−0.00 (−0.33)	0.31 (61.37)
*E* – *R*	0.39*	−0.05 (−12.48)	0.24 (63.04)	0.00 (0.83)	0.19 (48.61)

†SD: S – Species richness, E – Endemics richness. GD: Sh – Shannon diversity index, R – rarity index. Community diversity estimated from floristic data. Genetic diversity is estimated using AFLP markers collected from seven representative plant species of the Atlantic coast.

‡Pearson correlation computed among the diversity statistics (the effect of spatial autocorrelation is removed). Significance levels assessed using permutation tests (50 000 permutations, *P‐value <0.05).

§Correlation decompositions, according to Lamy et al. (2013). These terms represent the fraction of correlation between the SD and GD indexes that arises due to parallel effects of a given environmental variable on SD and GD. Because these metrics are computed on standardized variables, their sum equals to the Pearson correlation coefficient. For example, anthropogenic pressure (*St*) contributes for 0.15 correlation units – representing 29.81% of the total – of the correlation between the species richness (*S*) and *S*hannon diversity (*Sh*) indexes. The contribution of residuals represent the portion of SGDC that is not accounted by the considered explanatory variables.

Habitat disturbance varied among grid cells and differed markedly by origin. Natural habitat disturbance (*H*) was homogeneous throughout the study area, when averaged at the regional scale. In contrast, a clear hotspot of anthropogenic disturbance (*St*) was detected in the upper half of the study area and corresponded to grid cells with decreased GD–SD levels. Accordingly, the correlation decomposition analysis revealed that habitat disturbance accounted for 33–64% of the quantified SGDCs (Table 1), with the best explanatory power occurring in SGDCs that involved the rarity diversity index. Also, anthropogenic disturbance appeared as the leading contributor to all SGDCs. Congruent results were obtained by the RDA analysis, where the overall habitat disturbance (i.e., *H*,* St* and *St *×* H*) accounted for 34% of the variance shaping the joint GD–SD datasets (Table 2). Anthropogenic disturbance (*St*) accounted alone for 21% of the variance (Table 2, highly significant) and was significantly associated with decreasing diversity levels (Fig. 2B). In contrast, natural habitat disturbance (*H*) and its interaction with anthropogenic disturbance (*St *×* H*) accounted, respectively, for 5% and 8% of variance (Table 2) and did not explain GD–SD diversity patterns at a significant level (Fig. 2B). Importantly, between 36% and 67% of the SGDCs could not be explained by habitat disturbance (Table 1).

**Table 2:** Partial Constrained Redundancy analysis. We use a multivariate approach to test whether the natural dune dynamic (H) and the anthropogenic pressure (St), and their interaction (St* *×* *H) explain the joint species and genetic diversity patterns observed along the Atlantic coast. The effects of spatial autocorrelation are removed from the model

Variable†	df‡	Var (% Total)‡	*F* ‡	*P*‐value‡
*St*	1	0.85 (21)	4.85	0.01**
*H*	1	0.18 (5)	1.02	0.37
*St *×* H*	1	0.32 (8)	1.82	0.16
Model residuals	15	2.64		

†Model formula: Diversity ~ Environment | Geo. Where Diversity = complete set of diversity statistics (*S*,* E*,* Sh* and *R*), Environment = set of explanatory variables describing the natural dune dynamic (*H*) and the anthropogenic pressure (*St*), and their interaction (*St *×* H*) that act on every investigated grid cell. Geo = latitudinal projection of each grid cell (used as a covariable to remove the effect of spatial autocorrelation).

‡df, degrees of freedom; Var, variance and corresponding proportion of total variance explained by the focal variable; *F*, pseudo‐F statistic; *P*‐value, significance level, assessed with 50 000 permutations (**P‐value <0.01).

## Discussion

In agreement with previous studies (Vellend 2003, 2004; Cleary et al. 2006; Papadopoulou et al. 2011; Struebig et al. 2011; Blum et al. 2012; Wei and Jiang 2012; Lamy et al. 2013), our results show that a positive correlation between GD and SD can emerge; however, to our knowledge, this study is among the first to report a positive and significant correlation between SD and GD based on a multispecies approach at the regional scale. This correlation appears to be reasonably explained by varying levels of anthropogenic disturbance along the coastline, with more strongly disturbed areas characterized by reduced diversity and less disturbed sites characterized by greater diversity. However, only between 36% and 67% of the SGDCs (representing 21% of the variance present in the diversity dataset) could be traced back to a human impact. It is therefore likely that additional environmental parameters, such as local edaphic (e.g., richness in calcite carbonate, Granereau, pers. obs.) and meteorological conditions, as well as landscape connectivity (Lamy et al. 2013) might also account for the unexplained part of variance.

Here, anthropogenic disturbance is measured as the amount of urbanized surface within 10 km of the coastline. This proxy of anthropogenic disturbance may reduce SD and GD in two main ways: first, via habitat destruction through the construction of infrastructure (camp sites, holiday houses, golf courses, parking lots, etc.) that reduces available dune habitats (by perturbing their natural dynamics and eventually decreasing the width of the dune profile) and fragments the formerly continuous sand belt, and second, via habitat degradation around developed areas through trampling, littering, and eutrophication. Specifically, habitat destruction and degradation may reduce SD due to local extirpations of species and may reduce GD through increased genetic drift during population bottlenecks and founder events during recolonization. Additionally, within a fragmented and extremely narrow landscape such as a sandy coast, migration among habitat patches may be exacerbated, especially for plants that do not disperse via water currents, which may delay the restoration of GD and SD.

Studies of SGDC patterns in habitats disturbed by humans have yielded contrasting results. For instance, Wei and Jiang (2012) did not detect a correlation between the GD of a dominant tree species and SD in disturbed forests because the reduction of GD within disturbed sites was not paralleled by a reduction in the GD of the trees. On the other hand, Vellend (2004) reported a positive correlation between the GD of a common forest herb and the SD of the forest herb community, which was driven by higher GD and SD values in primary forest compared to secondary forest. These findings indicate that the outcome of SGDC studies may strongly depend on the choice of the focal species on which GD estimates are based because species differ in life‐history and functional traits (see Laroche et al. 2015 for a theoretical treatment of SGDCs). Hence, species may vary in their reaction to a factor hypothesized to drive SGDCs (Vellend and Geber 2005). Here, we tried to resolve this issue by estimating GD based on more than one species, but it is very likely that differences in susceptibility to disturbance among our focal species introduced some unexplained variation in the observed SGDCs. Also, although averaged diversity metrics can capture the effects of environmental factors that most strongly impact the landscape dynamics (as suggested by simulations, Box 1), species‐specific responses are likely to be ignored and remain unexplained.

The human impact on GD and SD is most pronounced in the center of the study region (between 44.2° and 44.8° north). Municipalities with the largest tourist accommodation capacities, resident population sizes, and amount of urbanized coastal landscape are located in this area (p. e. Lège‐Cap‐Ferret, Arcachon, La‐Teste‐de‐Buch, Biscarrosse and Mimizan). Favennec (2002b) and Forey et al. (2008) found a significantly lower species richness and endemism rates in the center of the study region. Increased human disturbance affecting coastal sand dunes around Mimizan and Lège‐Cap‐Ferret led to strong dune erosion in the 1970s and 1980s (Barrère et al. 1997; Duffaud et al. 1997). It has been shown in other parts of the French Atlantic coast that anthropogenic disturbance can lower the SD of sand dune communities for decades, even after successful dune stabilization and reestablishment of vegetation (Rozé and Lemauviel 2004).

Habitat disturbance may have particularly affected species endemic to the French Atlantic coast. Historical data suggest a larger and a more continuous distribution of endemics such as *Alyssum loiseleurii, Astragalus baionensis, and Hieracium eriophorum* during the 19th century until the first half of the 20th century, when increased waterside development started (e.g., Loiseleur–Deslongchamps 1806; Lloyd and Foucaud 1886; Jeanjean 1961; Frey et al. 2012a,b).

## Conclusions

In this study, we show that GD and SD within a coastal sand dune plant community can covary at the regional scale, and this pattern is at least in part driven by varying levels of human landscape alteration. Our study nevertheless suggests that evaluating additional drivers of the landscape diversity, notably those affecting the metapopulation and community dynamics (e.g., local connectivity, Lamy et al. 2013), is of importance. Seaside development and associated disturbances due to recreational use may have fragmented a formerly continuous sand dune habitat, most likely leading to the local extinction of species and the loss of GD due to population bottlenecks and founder effects. Species having large and continuous populations such as many dune species are especially prone to the loss of genetic diversity and genetic threats to survival when reduced in population size (e.g., Ellstrand and Elam 1993). Therefore, dune management policies should impede a further degradation and destruction of dune habitats.

## Supporting information


**Table S1.** Species included in the genetic diversity survey.
**Table S2.** AFLP datasets.
**Table S3.** Proxy data for human disturbance of the coastal sand dunes.
**Table S4.** Correlation decomposition, including non‐native and weedy species.
**Table S5.** RDA analysis, including non‐native and weedy species.
**Figure S1.** Principal Component Analysis of vegetation plots.
**Figure S2.** Genetic diversity estimated by the Shannon index and rarity index along the coastal gradient.
**Data S1.** R script implementing the correlation decomposition analysis.
