# Supplementary material for: Parallel declines in species and genetic diversity driven by anthropogenic disturbance: a multispecies approach in a French Atlantic dune system
Source: Evol Appl. 2016 Jan 27;9(3):479–88. doi: 10.1111/eva.12351 (PMC4778109; doi:10.1111/eva.12351)
Supplement: Supplementary file 1 — Table S1. Species included in the genetic diversity survey. Table S2. AFLP datasets. Table S3. Proxy data for human disturbance of the coastal sand dunes. Table S4. Correlation decomposition, including non‐native and weedy species. Table S5. RDA analysis, including non‐native and weedy species. Figure S1. Principal Component Analysis of vegetation plots. Figure S2. Genetic diversity estimated by the Shannon index and rarity index along the coastal gradient. Data S1. R script implementing the correlation decomposition analysis. [file EVA-9-479-s001.doc]

**Supplementary materials for:**

**Parallel declines in species and genetic diversity driven by anthropogenic disturbance: a multi-species approach in a French Atlantic dune system**


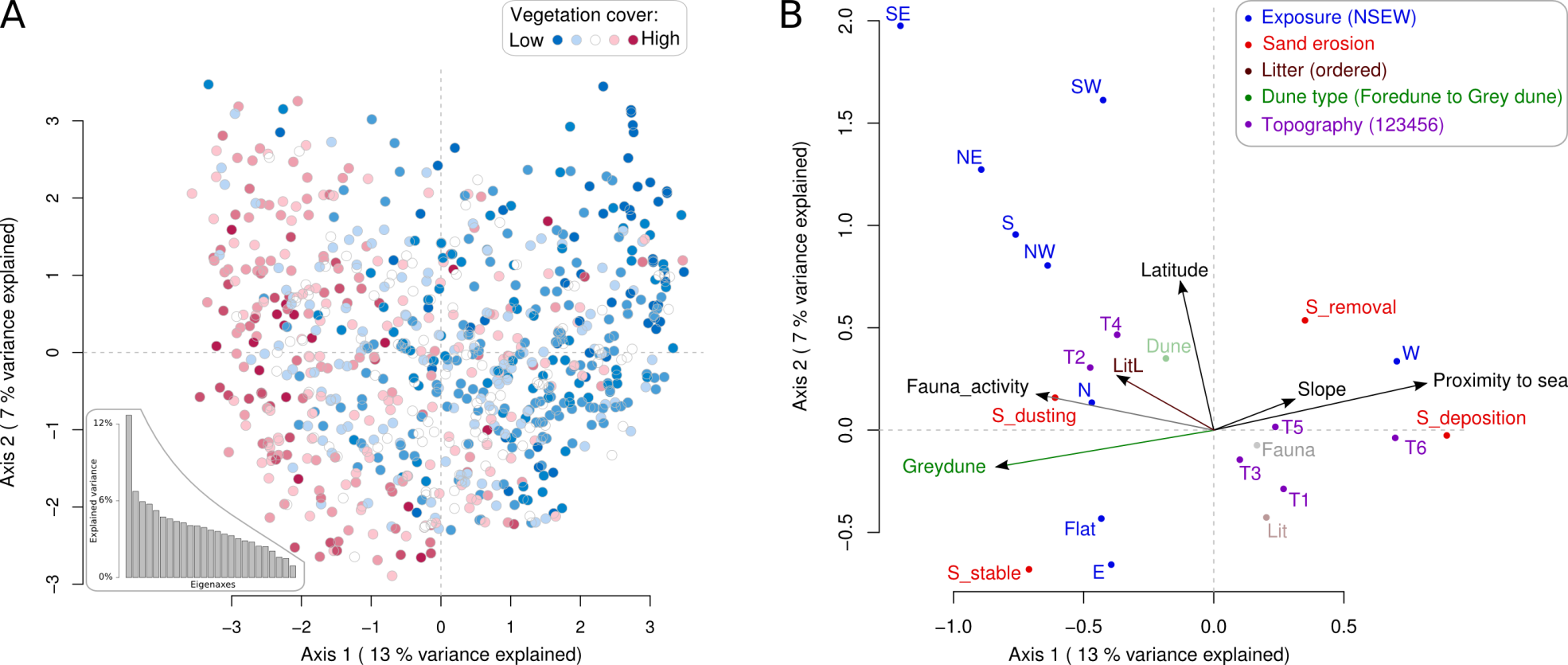


**Supplementary figure S1** Principal Component Analysis of 773 vegetation plots sampled by the French National Forest Office (ONF) characterized for eight habitat descriptors related to natural disturbance in coastal sand dune habitats: (i) dune facies, (ii) exposure, (iii) litter amount, (iv) microtopography, (v) rabbit activity, (vi) ranged distance of sampling plots from forest to ocean (vii), slope and (viii) wind perturbation regime. **A.** Vegetation plots, labeled according to percentage of soil covered by vegetation. Vignette: proportion of variance explained by each eigenaxis. **B.** Environmental descriptors. Note that the first eigenaxis (EIG1) correlated with the natural disturbance gradient, with positive values outlining disturbed plots (closer to ocean, with low vegetation cover) whereas negative values point towards stable areas (located closer to forest with high vegetation cover). In subsequent analyses, EIG1 is used as a proxy of natural habitat disturbance. Note that the linear contribution of latitude to EIG1 is removed before performing analyses presented in the main text. The present PCA is applied on a mixture of quantitative (position of sampling plot, slope), semi-quantitative (dune facies, fauna activity and litter) and qualitative variables (exposure and topography), using methods implemented in the ade4 R CRAN package (“dudi.mix” function). This approach replaces semi-quantitative variables by their orthogonal polynomials (with linear and quadratic components figured as arrows and dots, respectively).


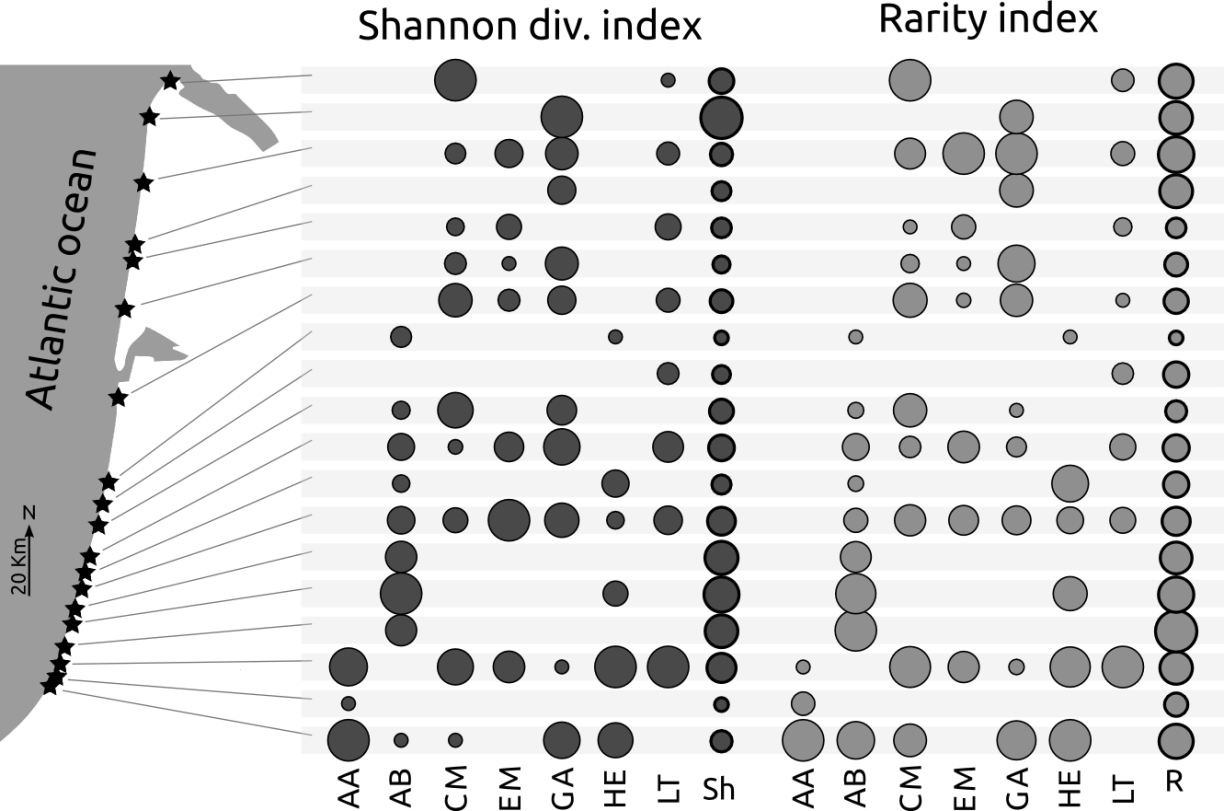


**Supplementary figure S2** Genetic diversity estimated by the Shannon index and rarity index along the coastal north-south gradient of seven typical and abundant plant species (AA, *Alyssum loiseleurii*, AB, *Astragalus baionensis*, CM, *Cakile maritima*, EM, *Eryngium maritimum*, GA, *Galium arenarium*, HE, *Hieracium eriophorum*, LT, *Linaria thymifolia*) of the sand dunes of the French Atlantic Coast. Species diversities are averaged at the sampling site level, after being standardized (missing data are ignored). The resulting composite indexes, *Sh* and *R*, are used in the main manuscript as descriptors of habitat genetic diversity. All species except *A. loiseleurii*, *A. baionensis* and *H. eriophorum* are continuously distributed throughout the study region. The latter occur mainly in the southern part, where they are locally abundant (van der Maarel and van der Maarel-Versluys 1996; Favennec 1998).

**Supplementary table S3** Proxy data for human disturbance of the coastal sand dunes including resident population census in 2009 (*Pop*), number of housings in 2009 (*Log*), amount of urbanized surface within 10 km coastline in 2006 (*St*) and tourist accommodation capacity in number of beds in 1999 (*Cht*). Data are provided by the French National Sea and Coast Observatory (*Obsérvatoire national de la mèr et du littoral*; ONML; available at <http://www.onml.fr/outil-de-cartographie/presentation-de-loutil/>)

| Postcode | Municipality | Latitude | Pop | Log | St | Cht |
| --- | --- | --- | --- | --- | --- | --- |
| 33544 | Le Verdon-sur-Mer | 45.544724 | 1334 | 1817 | 277.9 | 3621 |
| 33514 | Soulac-sur-Mer | 45.514168 | 2711 | 4901 | 555.76 | 21076 |
| 33193 | Grayan-et-l'Hopital | 45.445 | 1174 | 2383 | 407.87 | 15410 |
| 33541 | Vensac | 45.399445 | 854 | 690 | 29.01 | 2784 |
| 33540 | Vendays-Montalivet | 45.355831 | 2288 | 4279 | 579.93 | 21830 |
| 33300 | Naujac-sur-Mer | 45.254166 | 810 | 576 | 109.23 | 3362 |
| 33203 | Hourtin | 45.18528 | 3001 | 3361 | 420.46 | 15638 |
| 33097 | Carcans | 45.079445 | 2165 | 3755 | 418.62 | 19972 |
| 33214 | Lacanau | 44.977779 | 4412 | 8782 | 1108.65 | 38597 |
| 33333 | Le Porge | 44.873055 | 2428 | 2130 | 363.71 | 7445 |
| 33236 | Lège-Cap-Ferret | 44.797222 | 7527 | 10712 | 1550.55 | 47312 |
| 33009 | Arcachon | 44.658333 | 11441 | 16339 | 677.88 | 46202 |
| 33529 | La Teste-de-Buch | 44.632778 | 24597 | 15236 | 3278.75 | 24039 |
| 40046 | Biscarrosse | 44.395 | 12163 | 10207 | 2240.88 | 38596 |
| 40108 | Gastes | 44.324722 | 602 | 726 | 222.21 | 3497 |
| 40257 | Sainte-Eulalie-en-Born | 44.272778 | 1116 | 825 | 71.8 | 2859 |
| 40184 | Mimizan | 44.201111 | 7000 | 7457 | 942.32 | 25421 |
| 40266 | Saint-Julien-en-Born | 44.061943 | 1450 | 1598 | 263.81 | 8302 |
| 40157 | Lit-et-Mixe | 44.033611 | 1497 | 1603 | 286.45 | 8605 |
| 40326 | Vielle-Saint-Girons | 43.916668 | 1160 | 1197 | 325.08 | 13092 |
| 40187 | Moliets-et-Maa | 43.849724 | 888 | 3277 | 516.99 | 12052 |
| 40181 | Messanges | 43.815277 | 986 | 1131 | 234.09 | 14796 |
| 40328 | Vieux-Boucau-les-Bains | 43.787498 | 1577 | 3978 | 255.58 | 16608 |
| 40310 | Soustons | 43.75639 | 7240 | 5699 | 653.21 | 8275 |
| 40296 | Seignosse | 43.690277 | 3307 | 6483 | 554.57 | 27825 |
| 40304 | Soorts-Hossegor | 43.665833 | 3668 | 5134 | 721.58 | 15564 |
| 40065 | Capbreton | 43.646389 | 7864 | 9859 | 686.46 | 32013 |
| 40133 | Labenne | 43.59528 | 4644 | 2299 | 530.99 | 8739 |
| 40209 | Ondres | 43.562222 | 4479 | 2207 | 329.83 | 2970 |
| 40312 | Tarnos | 43.540279 | 11798 | 5768 | 982.79 | 960 |

*Analyses including non-native species*

Script S1

require(vegan)

CorDecomp = function(Y, X, Z){

### Input parameters:

# Y = two columns matrix (SG and SD)

# X = environmental predictors of interest

# Z = confound (a single variable), of which we remove the effect

### Output:

A list containing the following items:

out$Mainterms = SGDC contributions of the environmental predictors (matrix format).

out$Interactions = SGDC contributions of pairwise interactions of environmental predictors (matrix format, pred1:pred2, pred1:pred3, pred2:pred, etc).

out$SGDCglobal = Pearson correlation between SG and DC (corresponds to sum of out$MainTerms + out$Interactions).

out$PctDecomp = Summary statistics, showing global contributions (as %) of MainTerms, Interactions and Residuals to SGDCglobal.

### Script

# remove confounding effects of Z

TMP = cbind(Y, X, Z)

for(i in 1:ncol(TMP)){

tmp = residuals(lm(TMP[, i] ~ TMP[, ncol(TMP)]))

TMP[, i] = tmp

}

Y = TMP[, 1:ncol(Y)]

X = TMP[, (ncol(Y) + 1) : (ncol(TMP) - 1)]

# standardize everything

Y = decostand(Y, method = "standardize")

X = decostand(X, method = "standardize")

Z = decostand(Z, method = "standardize")

# compute correlation and covariance matrix among predictors

varsX = apply(X, 2, var)

covsX = cov(X)

corsY = cor(Y)

# regress Y on each X (multiple regressions)

# retrieve coefficients and residuals out of it

coeffs = NULL

resids = NULL

for(i in 1:2){

tmp = data.frame(Y = Y[, i], X)

vars = colnames(tmp)

form = formula(paste("Y ~", paste(vars[-1], collapse = ' + ' )))

model = lm(form, data = tmp)

coeffs = cbind(coeffs, coefficients(model))

resids = cbind(resids, residuals(model))

}

coeffs = coeffs[ -1,]

# compute covariance among residuals

covsResid = cov(resids)

# compute contributions of each predictor the the SGDC correlation

Rcontrib = function(i, j, ...){

sumAB = coeffs[i, 1] * coeffs[j, 2] + coeffs[j, 1] * coeffs[i, 2]

covsX[i, j] * sumAB

}

ix = 1:ncol(X)

pp=outer(ix[-1], ix[-length(ix)], function(ivec, jvec) sapply(seq(along =ivec),

function(k) {

i=ivec[k]

j=jvec[k]

if (i > j)

Rcontrib(i, j)

else NA

}))

rownames(pp) = colnames(X)[-1]

colnames(pp) = colnames(X)[-ncol(X)]

contribinteraction = pp

# produce outputs

contribpreds = apply(cbind(varsX, coeffs), 1, prod)

interct = sum(contribinteraction, na.rm = T)

rsds = cov(resids)[1, 2]

global = sum(contribpreds) + interct + rsds

list(MainTerms = data.frame(contribpreds),

Interactions = contribinteraction,

Residuals = rsds,

SGDCglobal = c(global, corsY[1, 2]),

PctDecomp = round(100 * data.frame(MainTerms = sum(contribpreds),

interct,

rsds) / global, 2))

}

### Usage:

# CorDecomp(Y, X, Z)

References

Favennec J. 1998. Guide de la flore des dunes littorales non boisées. Éditions sud ouest, Luçon.

Frey, D. J., Haag, C. R., Kozlowski, G., Tison, J.-M., & Mráz, P. (2012). High genetic and morphological diversity despite range contraction in the diploid Hieracium eriophorum (Asteraceae) endemic to the coastal sand dunes of south‐west France. Botanical Journal of the Linnean Society, 169(2), 365-377.

Maarel, E. v. d. and M. v. d. Maarel-Versluys. 1996. Distribution and Conservation Status of Littoral Vascular Plant Species along the European Coasts. Journal of Coastal Conservation **2**:73-92
